# Algorithmic management of postcardiotomy shock with mechanical support: Bring a map, a plan, and your parachute—and know how to use all three

**DOI:** 10.1016/j.xjon.2021.10.055

**Published:** 2021-11-02

**Authors:** Louis H. Stein, Scott C. Silvestry

**Affiliations:** a Northern Department of Cardiothoracic Surgery, RWJ-Barnabas Health, Newark, NJ; b Advent Health Transplant Institute, Orlando, Fla

**Keywords:** cardiogenic shock, ECLS treatment, ECMO treatment, heart-assist device, Impella, left ventricle decompression, mechanical circulatory support, ventricular assist device

**Figure:**
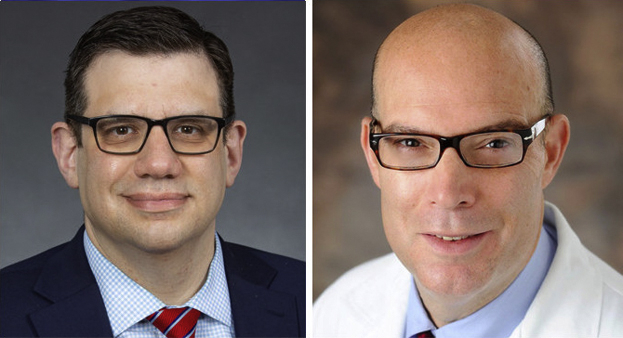
Drs Stein and Silvestry.

**Central Message**

Successful treatment of PCS shock begins with the preoperative assessment and requires a team-based approach. Mechanical support should be viewed as a valuable tool, not a mark of failure.

Adequate systemic perfusion is the primary goal for adult patients after surgery with cardiopulmonary bypass (CBP) for acquired cardiac disease. Depressed myocardial function, hypovolemia, and vasodilation are common and can contribute to decreased oxygen delivery. It is typical for patients to require some vasoactive or inotropic support in the initial postoperative period post-CPB. When post-CPB shock is refractory to medical intervention, it is generally referred to as “postcardiotomy shock” (PCS), a rare and potentially lethal condition.^1^ PCS typically manifests when the patient is unable to separate from CPB, but can progress later in the perioperative period. PCS is first addressed with pharmacologic support followed by insertion of an intra-aortic balloon pump (IABP).^2^ In recent years, there has been renewed interest in using mechanical circulatory support (MCS) for PCS.

The decision to proceed with MCS is based on patient risk factors, the nature of myocardial failure, and PCS severity. At times, this decision depends more on surgeon experience, biases, and device availability than other objective factors. The separation among acceptable, “typical” postoperative support, and true hemodynamic compensation is not easily defined.

Successful cardiac surgery requires assessing the patient's physiologic status and reserve, their disease, and the impact of the required operation. Preoperatively, risk factors for PCS should be identified and discussed as a multidisciplinary team with a consensus of intervention thresholds. Intraoperatively, the degree of shock can be clouded by surgeon bias and hopes that things will improve with time. For some, requiring MCS represents a surgical failure. In a pressured intraoperative setting, some team members may be reluctant to voice their assessment that MCS is required. A systematic, multidisciplinary approach, beginning with preoperative assessment, affords the best chance for an optimal outcome.

## Preoperative Assessment

“Plans are worthless, but planning is everything.”

Dwight D. Eisenhower

**Figure 1:**
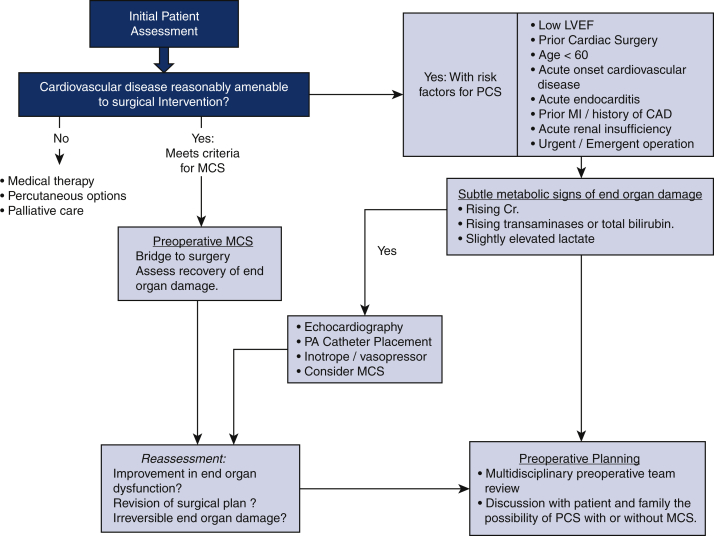
Preoperative assessment and planning. Patients who would benefit from preoperative MCS should be identified. Those at risk for PCS should be identified and a multidisciplinary plan of care formulated. *PCS*, Postcardiotomy shock; *LVEF*, left ventricular ejection fraction; *MCS*, mechanical circulatory support; *MI*, myocardial infarction; *CAD*, Coronary artery disease; *Cr*, creatinine; *PA*, pulmonary artery.

PCS is an infrequent complication that occurs in less than 4% of patients undergoing cardiac surgery.^3^ Accurate assessment is essential to determine a patient's risk for PCS potentially requiring MCS. Outcome predictors typically have not calculated the probability of PCS requiring MCS or categorized them accurately. Patient characteristics associated with PCS ultimately requiring MCS include a history of cardiac surgery, age less than 60 years, preoperative renal insufficiency, coronary artery disease with prior myocardial infarction, left ventricle (LV) dysfunction, acute onset of illness requiring urgent or emergency operation for acute myocardial infraction, left main coronary artery disease, and acute endocarditis.^4^ Such cases are often associated with intraoperative findings that require more complex surgery than expected. The optimal myocardial protection strategy for a given patient also should be planned. Despite the low likelihood of requiring MCS, the possibility for and thresholds to implement MCS are important considerations (Figure 1).

### Patient-Specific Issues

To plan for potential MCS support of PCS, the team should discuss patient-specific risk factors, expected problems, and support strategies. Although not a comprehensive list, some of the factors to consider include the following:

• Preoperative right ventricle (RV) dysfunction: Consider RV optimization with diuresis, milrinone, or dobutamine. The team plan should include possible postoperative right ventricular assist device (RVAD) or venoarterial extracorporeal membrane oxygenation (VA-ECMO).
• Peripheral vascular disease: Consider limitations to axillary or femoral artery access. In severe cases, the CPB outflow cannula may need to be used for MCS.
• Mechanical aortic valve: Prohibits transaortic support options.
• High bleeding risk while on MCS in patients with inherited coagulopathies or recent use of anticoagulants.

If PCS is considered possible, a frank discussion with the patient and family should be initiated. Possible end points and best-case and worst-case scenarios should be discussed, ensuring consistency with the patient's goals of care. They should address possible outcomes; recognizing the inability to wean may be a possibility. Discussing potential options with the surgical team during operative briefing aids preparation.

### Scoring Systems

Quantifying a patient's PCS risk is difficult. Cardiac surgery risk calculators, such as The Society of Thoracic Surgeons and European System for Cardiac Operative Risk Evaluation II, do not directly calculate the probability of PCS. The SAVE and ENCOURAGE scores have demonstrated utility in predicting MCS outcomes for patients in cardiogenic shock, but are not validated for patients with PCS.^5^ The REMEMBER score, derived from a cohort after coronary artery bypass grafting, may have some utility, although broadened validation is still required.^6^ Although none of these models ideally predict PCS or the need for ECMO, they add valuable perspective by appraise surgical risk.

### Subjective Bias

Executing MCS for PCS is rare and frequently unexpected. Decision fatigue and surgical bias may delay diagnosing PCS. Many may consider a patient on “maximum drips” preferable to using MCS, an outcome they perceive as a technical failure. Hoping “the patient will get better with time” may lull the surgeon into witnessing the progression of cardiogenic shock. Conversely, some patients with preoperative hemodynamic instability or low ejection fraction may be deemed to be “too sick” to undergo surgery. These patients may benefit from a surgery in which MCS is a component.

## Preoperative Device Insertion

“Begin with the end in mind.”

Stephen Covey

Preoperative shock is a significant risk for PCS. Medical treatment of low cardiac output may be inadequate in acutely decompensated patients. Preoperative MCS may facilitate optimizing these patients by augmenting end-organ perfusion and reversing organ injury. Enhanced perfusion with an IABP or percutaneous left ventricular assist device (LVAD) inserted via an axillary artery may improve renal function and reverse metabolic derangements, while allowing ambulation and providing meaningful improvement to the patient's risk profile.^7^ Some patients presenting with hemodynamic decompensation and an indication for emergency surgery will benefit from preoperative resuscitation and stabilization on VA-ECMO. Hemodynamic stability provided by preoperative MCS allows time to determine whether end-organ damage is reversible. Continued or worsening shock liver, acute kidney injury, or lactic acidosis demonstrate that end-organ damage is irreversible and the futility of further intervention. Myocardial ischemia and ischemic ventricular septal defect are some examples where this practice has been demonstrated with excellent results.^8^^,^^9^

## Operative Decision-Making

“Everyone has a plan until they get punched in the mouth.”

Mike Tyson

**Figure 2:**
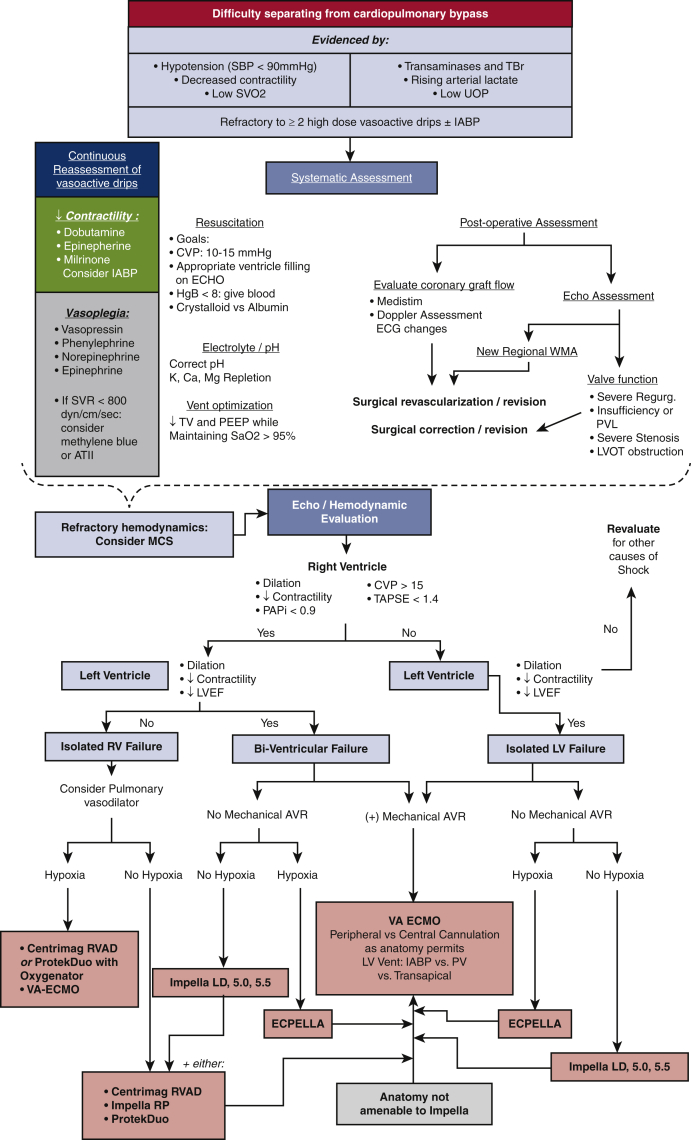
Intraoperative algorithm for the institution of MCS in patients with cardiogenic shock. A systematic evaluation for and correction of mitigating factors is performed. Once refractory PCS is identified, a hemodynamic and echocardiographic evaluation is performed to determine optimal strategy. *SBP*, Systolic blood pressure; *SVO2*, mixed venous saturation; *UOP*, urine output; *IABP*, intra-aortic balloon pump; *SVR*, systemic vascular resistance; *ATII*, Angiotensin II; *CVP*, central venous pressure; *ECHO*, echocardiogram; *ECG*, electrocardiogram; *WMA*, wall motion abnormality; *PVL*, paravalvular leak; *TV*, tidal volume; *PEEP*, positive end-expiratory pressure; *LVOT*, left ventricular outflow tract; *MCS*, mechanical circulatory support; *PAPi*, pulmonary artery pulsatility index; *TAPSE*, tricuspid annular plane systolic excursion; *LVEF*, left ventricular ejection fraction; *RV*, right ventricle; *LV*, left ventricle; *RVAD*, right ventricular assist device; *VA-ECMO*, venoarterial extracorporeal membrane oxygenation; *TBr*, total bilirubin; *AVR*, aortic valve replacement; *PV*, pressure volume.

The inability to maintain adequate systemic perfusion despite 2 inotropes while attempting to separate from CPB is typically the first manifestation of PCS. An appropriate preoperative plan, executed systematically, based on objective data provides the best chance for optimal outcomes (Figure 2).

### Initial Management of Postcardiotomy Shock

It is commonplace to require some pharmacologic support when separating from CPB, which can obscure the recognition of PCS. In general, PCS presents with hypotension, depressed contractility, and ventricular distention, and increasing doses of several drips are common. Once it is recognized the postoperative heart cannot support systemic perfusion, the team must perform an expeditious evaluation of possible mitigating factors.

### Postbypass Checklist

Multiple factors can contribute to severe postoperative myocardial dysfunction. Correction of these may improve myocardial function and the efficacy of non-MCS interventions. A mnemonic device may help: “R-AVERAGED”: **R**esuscitation **- A**cidosis, **V**alve function, **E**lectrolytes, **R**hythm, **A**irway (ventilation), **G**raft function, **E**CG changes/wall motion abnormalities, **D**rips can structure a systematic review of contributing factors (Table E1).

### Threshold

The decision to implement MCS is a team-based synthesis of objective data rather than gestalt. Using established criteria such as the Columbia University Protocol (Table 1)^10^ or other indicators such as elevated filling pressures or a cardiac index less than 2 L/min/m^2^ despite 3 high-dose pressers assists decision-making.^11^ Doses of inotropes and vasopressors can be increased until predetermined thresholds are reached. A “wait and see” approach may miss the window for recovery, permitting end-organ damage to progress. Several studies on patients with cardiogenic shock demonstrated a correlation between earlier initiation of VA-ECMO and improved survival (Table E3). For PCS, cannulation at the time of surgery is associated with better outcomes than cannulation later in the intensive care unit.^10^

**Table 1:** Adapted Columbia University Criteria to prompt consideration of mechanical circulatory support for postcardiotomy shock

Clinical indicators of low perfusion	• Low SVO_2_ • Rising arterial lactate • Low UOP	
In the setting of		
Refractory to vasoactive medications.	≥2 high-dose inotropes or ≥2 high-dose vasopressors	Examples of high-dose drips: • Norepinephrine >10 μg/min, • Epinephrine >4 μg/min • Dobutamine >5 μg/kg/min

*SVO2*, Mixed venous oxygen saturation; *UOP*, urine output.

### Assessment of Ventricular Failure

The importance of accurately identifying the underlying deficits causing hemodynamic compromise cannot be understated. Medical management, device choice, and prognostication of recovery are each influenced by the etiology. Characterization of myocardial dysfunction should be based on a synthesis of echocardiographic and hemodynamic data, as well as clinical insight. Transesophageal echocardiography (TEE) is an invaluable tool for assessing LV and RV function. A review of intraoperative TEE assessment is beyond the scope of this opinion article and can be found in Table E3. Hemodynamic measurements from a Swan-Ganz catheter are considered essential in augmenting assessment of loading conditions, pulmonary hypertension, and ventricular function. For example, the pulmonary artery pulse pressure normalized to right atrial pressure or pulmonary artery pulsatility index less than 0.9 is a reliable indicator of declining RV function.^12^ On the basis of these findings, the team should have a clear understanding if the patient is in LV, RV, or biventricular failure.

### Choice of Mechanical Circulatory Support Strategy

MCS has been used for PCS since the 1960s.^13^ Since then, the devices at the surgeon's disposal have evolved significantly. The choice of MCS is nuanced, and device availability, institutional expertise, and patient-specific factors should be considered. In general, we favor an approach that minimizes invasiveness and favors patient mobility with minimal impact on the systemic circulation.

An IABP is easily inserted, augments diastolic coronary flow, and reduces afterload. Its utility for PCS is limited given its modest ability to improve systemic flow.^2^ Extrapolation of the results from the IABP-SHOCK II trial has led some to question its utility in PCS.^14^ The IABP is a valuable adjunct to VA-ECMO by facilitating LV decompression.

VA-ECMO is ubiquitously available, making it a popular option for PCS. Central and peripheral configuration for the outflow and venous drainage are possible. No consistent data have favored central over peripheral over peripheral strategies.^15^^,^^16^ Multiple permutations of ECMO cannulation techniques are possible and should be adapted to patients' unique physiology and anatomy.^17^

The Impella (Abiomed) is a temporary, percutaneous, transaortic LVAD. The Impella LD, 5.0, and 5.5 are considered most appropriate in supporting cardiogenic shock. Survival was 57.6% among highly selected patients with PCS supported with the Impella 5.5.^18^ Peripheral arterial insertion may predispose patients to vascular complications. A comparison of features to consider when deciding between a VA-ECMO and Imella device are listed in Table E2.

Three options exist for RV support: the CentriMag (Abbott Laboratories), Protek Duo (TandemLife), and Impella RP (Abiomed). As an RVAD, the Centrimag drains blood from the cava or right atrium, with outflow to the pulmonary artery, providing maximum flows of approximately 5.4 L/min. Tunneled lines are necessary if chest closure is planned.

The Impella RP is placed via the femoral vein with the impeller positioned in the right atrium/inferior vena cava junction and the outflow in the pulmonary artery. The Protek Duo is an alternative percutaneous option for RV support. It uses a dual-lumen catheter, one for drainage in the right atrium and the other for outflow in the PA, providing up to 4.5 L/min of flow. An in-line oxygenator can be placed with the Centrimag or Protek Duo.

### Adjunct Therapy

Adjuncts to MCS for PCS should support the essential goals of allowing the myocardium to rest while permitting end-organ perfusion. The sprained ankle, which requires both rest and gentle range of motion to recover from injury, is a common analogy for treating injured myocardium. Supporting peripheral perfusion and ventricular decompression provide myocardial rest. We suggest the use of a low dose of inotrope to promote contractility (ie, mobility) during recovery while limiting stasis contributing to thrombus formation.

#### Left ventricular decompression

Adequate ventricular decompression is an essential consideration. LV decompression reduces myocardial strain, improves myocardial perfusion, and correlates with improved outcomes.^19^^,^^20^ LV decompression prevents acute lung injury, which significantly affects survival.^21^

The Impella allows efficient LV decompression and has been shown to be a valuable adjunct to ECMO or “ECPELLA,” improving survival by 21% over VA-ECMO alone.^22^ The IABP facilitates LV decompression with VA-ECMO, also demonstrating improved survival.^23^ Common central approaches for LV decompression include via the right superior pulmonary vein or the LV apex. These drainage cannulas are Y-ed to the systemic venous drainage, with an adjustable clamp to titrate the flow of the LV limb.

#### Right ventricular failure

MCS for isolated RV failure should be supplemented with dobutamine epinephrine or milrinone. Because of its lusitropic effects and reduction of pulmonary vascular resistance, MCS is the first choice for many. Some advocate empiric use of pulmonary vasodilators with RV failure.^24^ Given the likelihood of RV distention and myocardial edema, delayed chest closure should be considered.

#### Volume status and vascular tone

Achieving a fine-tuned balance between fluid status and vascular tone is critical in treating patients in shock. Peripheral vasodilation, or vasoplegia, is the manifestation of low systemic resistance and may cloud the picture hypotension with low cardiac output. Moderate doses of vasopressors can be used judiciously to maintain tone, keeping in mind the resulting increased afterload and microvascular ischemia. Refractory vasoplegia is typically addressed with agents such as high-dose vitamin B12, methylene blue, or angiotensin II.^25^ Adrenal insufficiency should also be considered.

Fluid resuscitation may be required in the initial postoperative period. As a rule, volume should be administered cautiously to prevent RV overload, myocardial edema, and third spacing. In the operating room, fluid resuscitation should be performed with the careful guidance of TEE and hemodynamics. Adequate preload is required for optimal myocardial function. Factors such as thickened myocardium indicate a higher than anticipated preload is required. Over-resuscitation may drive the heart “too far right” on the Starling curve, resulting in myocardial dysfunction.

Postoperatively, after the resuscitation period, the goal central venous pressure should be less than 15 mm Hg, and in many cases less than 10 mm Hg. Diuresis is typically required. Keep a low threshold for using temporary hemodialysis or Aquapheresis if sufficient pharmacologic diuresis cannot be achieved. A low-dose vasopressor is justified to facilitate fluid removal.

## Postoperative Decision-Making

As with any critically ill patient, vigilant monitoring, frequent reassessment, and adjustments are essential. Key elements of monitoring the patients in PCS include adequate end-organ function, neurologic status, extremity perfusion, and LV decompression. In addition to imaging and serial laboratory assessment, a collaborative intensive care unit team with clear communication and goals is ideal in the care of these acutely ill patients. Table 2 presents critical factors to monitor after MCS during the resuscitation phase.

**Table 2:** Common issues suggesting inadequate perfusion or mechanical circulatory support–related complications

Factors	Measures	Causes	Evaluation/plan
		• Insufficient perfusion (MAP or CO)	Insufficient cardiac output flow: Increase device flow. • Switch to device with higher flow ability. Calculate SVR: • Vasopressors as needed.
End-organ perfusion	Daily laboratory Evaluation: • Lactate :↑ • SVO2: ↓ • Creatinine: ↑; UOP: ↓ • Transaminases: ↑ • Bilirubin: ↑	• Venous congestion	CVP:↑ Echocardiography: RV or IVC distention. CXR: congestion • Aggressive diuresis • If ECMO adjust venous cannula
		• Thromboembolic disease	Targeted evaluation guided by clinical evidence of organ dysfunction. CTA evaluation for obstruction to end-organ flow. Evaluate for clot on echocardiography. Daily evaluation of neurologic status as possible. Doppler assessment of extremity perfusion
Ventricular decompression	Daily echocardiography	• Fluid overload. • Increased afterload.	Titrate device flow with echocardiographic guidance. Augment LV ventilation technique. Aggressive diuresis.
Extremity ischemia	Daily laboratory evaluation: Lactate :↑ CK: ↑ Physical exam	• Cannula obstructing distal flow. • Thromboembolic disease.	Imaging to evaluate flow. Placement of distal perfusion cannula. Embolectomy if indicated. Low threshold for fasciotomy.
Pulmonary congestion	Daily CXR Increasing requirements on ventilator/ECMO FdO2 and Sweep.	• Fluid overload. • Congestion with lack of LV decompression.	• Volume removal. • LV vent revision.

*SVO2*, Mixed venous saturation; *UOP*, urine output; *MAP*, mean arterial pressure; *CO*, cardiac output; *SVR*, systemic vascular reistance; *CVP*, central venous pressure; *RV*, right ventricle; *IVC*, inferior vena cava; *CXR*, chest x-ray; *CTA*, computed tomography angiography; *LV*, left ventricle; *CK*, Creatine Kinase; *ECMO*, extracorporeal membrane oxygenation; *FdO2*, fractional delivered fractional oxygen percentage.

### Special Considerations

Certain scenarios for PCS deserve special consideration when formulating an MCS strategy. Intracardiac thrombus can occur even in optimally anticoagulated patients.^26^ Low intracardiac flow permits stasis and thrombus formation. Valve repairs and prosthesis are notable locations for thrombus formation.^27^, ^28^, ^29^ Evaluation for intracardiac thrombus should be part of the regular echocardiographic evaluation.^26^

### Early Support and Weaning

In the early phase of support (typically 12-72 hours), the primary focus should be ensuring adequate end-organ perfusion and ventricular decompression. Once laboratory results normalize and intrinsic pulsatility above support indicate myocardial recovery, formal weaning trials can be considered.^27^ Daily trials are performed by reducing device support gradually to 2 L/min. Hemodynamics and echocardiography should be evaluated. Satisfactory hemodynamics without ventricular distention and adequate contractility while on a moderate-dose vasopressor indicate a patient is ready to be separated from MCS. The team should review the findings of each weaning trial and determine if modification of therapeutic strategy is needed.

### Exit Strategies and Goals of Care

Despite improvements in support options and overall prognosis, the most common outcome is death.^3^^,^^4^^,^^10^^,^^11^ From the outset, clear communication with the family is essential to set clear realistic expectations, timelines, and likely outcomes in family-oriented communication. Caregivers must balance the preclinical state, chronic health issues, and predicted clinical trajectory in the context of the patient's advance directives and family requests. In general, we establish at the time of starting MCS that the absence of significant improvement within 7 to 14 days portends a poor prognosis.^30^

Recognition of institutional limitations in caring for these patients is essential. For institutions without long durable MCS or transplant capabilities, establishing relationships with centers possessing these programs is indispensable for both guidance and possible transfer for advanced therapies such as LVAD or transplant.

Many patients with PCS will not survive despite exceptional care.^3^ In most recent studies, survival to hospital discharge ranges from 16% to 56%.^3^^,^^6^^,^^12^ When available, a palliative care team should be involved in the care of every patient requiring MCS for PCS. They are valuable additions to the acute team's efforts. Providing clear communication and establishing an environment for families to limit or stop further care are essential to partnering in the care. Establishing clear communication of progress, complications, and prognosis may be difficult in some circumstances, leading to prolongation of futile care.

## Conclusions

PCS portends a significant risk of mortality.^3^^,^^6^^,^^12^ Early use of MCS can significantly improve recovery chances.^9^^,^^10^ The odds of a successful patient recovery are improved through identification of patients at risk, multidisciplinary formulation of a preoperative plan, prompt initiation of support, and vigilant postoperative care. Family support, recognition of patient goals of care, and medical futility are equally essential.

### Conflict of Interest Statement

S.C.S. reports honoraria from Abbott, Medtronic, Syncardia, and Abiomed. L.H.S. reported no conflicts of interest.

The *Journal* policy requires editors and reviewers to disclose conflicts of interest and to decline handling or reviewing manuscripts for which they may have a conflict of interest. The editors and reviewers of this article have no conflicts of interest.

## Figures and Tables

**Table E1:** R-AVERAGED mnemonic for evaluation of myocardial dysfunction after cardiopulmonary bypass

**R**esuscitation	Careful fluid resuscitation should be conducted with the guidance of TEE and measured hemodynamics. Adequate preload is required. Excessive volume can push the ventricles too far right on the Frank Starling Curve, resulting in “overload.” The RV is particularly sensitive to this.
**A**cidosis	Check pH and correct to physiologic range.
**V**alves	Evaluate valvular function on TEE. Consider addressing new stenosis, regurgitation, or paravalvular leak with repair or replacement. Systolic anterior motion can be troublesome, particularly after mitral valve repair, and the struts of a mitral valve replacements can cause an LVOT obstruction.
**E**lectrolytes	Correct potassium/magnesium/calcium levels.
**R**hythm	Sinus rhythm is ideal, atrial “kick” provides 20% of ventricular filling. The rate should be fast enough to provide cardiac output but also allow enough time for ventricular filling.
**A**irway	Minimize PEEP and tidal volume to facilitate blood return, while maintaining appropriate gas exchange.
**G**raft	Coronary artery bypass grafts should be evaluated for patency and adequate flow. Doppler or transit time flow measurement can be particularly useful. If any grafts are in question, revision should be considered.
**E**CG	ECG changes such as ST elevation and new wall motion abnormalities indicate areas of poor perfusion due to obstructed or injured native coronaries or grafts. In such situations, the surgeon should have a low threshold for revascularization.
**D**rips	Vasoactive drips should be individualized to the patient's specific hemodynamics.

*TEE*, Transesophageal echocardiography; *RV*, right ventricle; *LVOT*, left ventricular outflow tract; *PEEP*, positive end-expiratory pressure; *ECG*, electrocardiogram.

**Table E2:** Commonly available mechanical circulatory support options for left ventricular and biventricular support

	VA-ECMO	Impella LD, 5.0, 5.5 (Abiomed)
Support	Biventricular	LV
Cannulation	Venous and arterial may be central or peripheral.	Femoral or axillary artery: 5.0/5.5 Direct aortic cannulation: LD/5.0/5.5
Oxygenation capability	Yes	No
Limb ischemia risk	With axillary or femoral artery cannulation. Distal perfusion cannula recommended. Consider monitoring extremity perfusion with NIRS.	
LV decompression	LV ventilation may be required, particularly with peripheral VV-ECMO.	Yes
Patient ambulation	Can be limited with femoral cannulation	
Thromboembolism risk	Yes	

*VA-ECMO*, Venoarterial extracorporeal membrane oxygenation; *LV*, left ventricle; *NIRS*, near-infrared spectroscopy; *VV-ECMO*, venovenous extracorporeal membrane oxygenation.

**Table E3:** Commonly available temporary right ventricular mechanical circulatory support devices

Device	Impella RP (Abiomed)	Protek Duo (TandemLife)	CentriMag RVAD (Abbott Laboratories)
Cannulation	Percutaneous femoral vein	Percutaneous right IJ	Central: RA to PA
Oxygenation capability	No	Yes	Yes
Risk of limb ischemia	No	No	No
Patient ambulation	Limited	Yes	Yes

*RVAD*, Right ventricular assist device; *IJ*, internal jugular vein; *RA*, right atrium; *PA*, pulmonary artery.

**Table E4:** Suggested additional reading

Lead author	Year	Journal
Intraoperative TEE evaluation		
Reeves^E1^	2013	*J Am Soc Echocardiogr.*
Nicoara^E2^	2020	*J Am Soc Echocardiogr.*
Effect of increased vasoactive dose and delayed MCS cannulation on outcomes in cardiogenic shock		
Samuels^E3^	1999	*J Card Surg.*
Lee^E4^	2021	*JACC Cardiovasc Interv.*
Management of anticoagulation		
Beavers^E5^	2021	*Pharmacotherapy.*
Rivosecchi^E6^	2021	*Crit Care Med.*
Management of SAM		
Varghese^E7^	2012	*J Thorac Cardiovasc Surg.*

*TEE*, Transesophageal echocardiogram; *MCS*, mechanical circulatory support; *SAM*, systolic anterior motion.
